# Comments on “Bridging Tumorigenesis and Therapy Resistance With a Non-Darwinian and Non-Lamarckian Mechanism of Adaptive Evolution” 

**DOI:** 10.3389/fonc.2021.775723

**Published:** 2021-12-13

**Authors:** Mesut Tez

**Affiliations:** Department of Surgery, Ankara Numune Hospital, Ankara, Turkey

**Keywords:** cancer, evolution, darwin, Lamarck, biology

I have read the hypothesis article entitled “Bridging Tumorigenesis and Therapy Resistance With a Non-Darwinian and Non-Lamarckian Mechanism of Adaptive Evolution” with great interest. I agree with the authors that cancer is an adaptation mechanism to the hostile microenvironment (1, 2). However, there are some points that must be clarified.

The authors stated that according to the “*use-it* or *lose-it* model”, positive selection is not necessary for cancer evolutionary adaptation (1). However, positive selection is the underlying mechanism of childhood cancers. Childhood tumors with small mutational burdens typically develop along several evolutionary trajectories within a single tumor. For example, the evolutionary natural history of childhood acute lymphoblastic leukemia (ALL) is almost completely hidden, clinically silent, and well advanced at the point of diagnosis. In patients with childhood acute lymphoblastic leukemia, prenatal or the first “hit” is very common, exceeding the clinical rate of ALL by about 100-fold, and indicates a low rate of penetration or evolutionary progression. Achieving critical secondary gene copy number variations requires some Darwinian selective advantage to increase the number of cells at risk, and the cytokine TGF beta may serve this function. The clonal architecture of ALL has been investigated by single cell analysis with multicolor probes for mutant genes. According to the available data, there is not a linear sequence of mutation acquisition by clonal succession but considerable complexity with a tree-like or branching structure of genetically distinct subclones, very reminiscent of Darwin’s original 1837 evolutionary divergence diagram (3).

Major advances in the field of transgenerational epigenetic inheritance have reopened a debate on the validity of Lamarck’s original theory that species may adapt phenotype in response to environmental influences (4). It is only recently that studies in mammals have provided evidence that exposure to environmental stressors can drive stably inherited phenotypic adaptations in offspring that are inherited by epigenetic rather than genetic mechanisms. Intriguingly, some of these studies concern the development of liver disease (5). Viral-etiology tumors can provide an understanding of the relationship between epigenetic dysregulation and cancer biology. Chimeric mice with humanized livers showed time-dependent, genome-wide changes in DNA methylation after being intravenously administered HBV or HCV. Viruses can also directly alter epigenetic reprogramming linked to hepatocellular carcinoma. Importantly, a number of common genes were methylated in this mouse model when compared with human HCC samples, indicating that it may be an important tool to investigate clinically relevant virus-induced epigenome remodeling (6). Briefly, it can be stated that hepatocellular carcinoma is in good agreement with the Lamarckian/quasi Lamarckian evolution (7). Thus, while the question of whether tumor evolution is predominantly driven by Darwinian or non-Darwinian selection remains open to discussion, childhood cancers can be explained by Darwinian evolution and sporadic cancers by Lamarckian/quasi Lamarckian evolution.

The atavistic model of cancer represents an atavism at the cellular level, and cancer cells are not just “rogue” cells generated through a series of random mutations but rather an ancient form of life that lies dormant within healthy metazoan cells. The atavistic cancer theory cannot be correlated with Darwinian or non-Darwinian evolution (8). The evolutionary basis of the atavistic theory may be the Brooks–Wiley evolution theory. According to the Brooks–Wiley theory, the dynamics of evolution derive from historically constrained increases in the information and entropy of a system of imperfectly reproducing organisms (8).

Heidegger considered Heraclitus of Ephesus (around 535–475 B.C.) along with Anaximander and Parmenides to be among primordial (anfänglich) philosophers (more precisely, the “thinking thinkers”) (9). Born into an aristocratic family, Heraclitus achieved lasting philosophical and theological relevance with his dynamic conception of physical reality and his logos doctrine. Being related to the verb legō, meaning I relate, speak, or say, the noun logos primarily means the word by which the inward thought is expressed and also the inward thought or reason itself. From this basis, further meanings of ‘word’, ‘story’, or ‘reason’ are derived. From Fragments 1 and 2, we can deduce the meaning of the logos for Heraclitus, namely, that it is constant and unfolds as the ‘together’ in beings, and everything that happens is in accordance with this constant ‘together’.

It appears that Heraclitus conceived the logos as providing a hermeneutical key for understanding the whole of reality. Understanding the logos is, therefore, the most important of all human activities (10, 11).

In brief, there is no single theory of evolution to explain carcinogenesis, and this probably indicates the necessity of a new theory of evolution (logos) rather than carcinogenesis. The model proposed by the authors (1) does not seem to be sufficient to explain childhood cancers, but it does bring a different perspective to the issue.

## Author Contributions

The author confirms being the sole contributor of this work and has approved it for publication.

## Conflict of Interest

The author declares that the research was conducted in the absence of any commercial or financial relationships that could be construed as a potential conflict of interest.

## Publisher’s Note

All claims expressed in this article are solely those of the authors and do not necessarily represent those of their affiliated organizations, or those of the publisher, the editors and the reviewers. Any product that may be evaluated in this article, or claim that may be made by its manufacturer, is not guaranteed or endorsed by the publisher.
